# The Global Burden of Food Insecurity Due to COVID-19

**DOI:** 10.3390/nu14173582

**Published:** 2022-08-30

**Authors:** Giuseppe Grosso

**Affiliations:** Department of Biomedical and Biotechnological Sciences, University of Catania, 95123 Catania, Italy; giuseppe.grosso@unict.it

The insurgence of the COVID-19 pandemic has demonstrated that no country or region was prepared to face such a devastating emergency, nor have researchers uncovered permanent solutions to solve this everlasting crisis thus far. Another clear evidence that has emerged over the last 2 years is the weak state of equilibrium under which the fragile and vulnerable populations are living while being at higher risk of succumbing to the worsening of previous conditions due to COVID-19. While clinical conditions, such as cardiorespiratory diseases, have been established to substantially increase mortality rates and those suffering from obesity have been found to have an increased risk of infection and worsening of all measured outcomes (hospitalization, undergoing UTI, and mortality rates), to the extent to which the current situation can fall under the definition of a syndemic, further attention has been put on the outcomes related to social conditions associated with COVID-19 [1]. A fervid share of scientific production has been produced over the very last period concerning food insecurity caused by the pandemic. Food insecurity measures include physical access, availability, and affordability due to the impact of the pandemic on employment [2]. The impact of COVID-19 restriction measures may have had a different impact depending on timing, duration, stringency, and different population groups (i.e., sex, age, socioeconomic status, etc.) [3]. Certain studies also underlined a potential impact of geographical location on food security, indicating that rural areas potentially present a higher risk of food unavailability and lack of access, in addition to facing lower diet quality and reduced food safety [4]. Limited access to food by any cause may have ultimately led to reduced ability to consume balanced meals meeting dietary and nutritional requirements during the pandemic [5]. Overall, the recent estimates from the World Food Programme aggravated by the Russian conflict in Ukraine show that up to 323 million people worldwide could become acutely food insecure by 2023 [6].

There is growing evidence that people living in poverty and racial and ethnic minorities are at higher risk of food insecurity as a result of COVID-19. In this regard, Niles et al. [7] explored the level of food insecurity in a sample of nearly 4000 individuals living in the US. The study showed a substantial increase (nearly 25% of interviewed) in food insecurity since the COVID-19 outbreak; the factors contributing to higher odds of food insecurity during COVID-19 were job loss, experiencing a furlough, or loss of hours, but also simply having more children, being a woman, and lower education. Another survey conducted in the US reported that 36% of low-income adults in the US were food secure, 20% had marginal food security, and 44% were food insecure [8]. This issue has been suggested to be even more challenging when considering children from low-income families with no access to school meals [9]. This topic gained much interest over time, and additional reports have been produced with updates all around the globe. A study investigating food insecurity in 653 households in Wuhan via an online questionnaire in March 2020 reported that more than 50% of the households had a Household Food Insecurity Access Scale Score of less than 9, indicating that lockdown measures had a huge negative impact on household food security compared with normal conditions [10]. A study from the UK Understanding Society COVID-19 Survey including nearly 10,000 individuals reported that approximately 13% of the sample reported someone in the household being unable to eat healthy or nutritious food, but only 2% reported being hungry and not eating; among factors associated with food insecurity, single parents and young people aged 16–30 years had a higher odds of reporting food insecurity [11]. A study conducted on 5811 participants living in Italy showed that among the families interviewed, the risk of food rose from about 8% before the pandemic to 16.2% after the pandemic began, with households from southern Italy being more at risk [12]. Data from Australian surveys have been gathered in some reviews concluding that food insecurity has risen during COVID-19 by about 20% compared with the conditions before the pandemic in both urban and rural/aboriginal communities, with roughly up to 10% of the respondents experiencing severe food insecurity (depending on the area of living) [13]. Other studies providing data on low- and middle-income countries from the African (multi-country sub-Saharan Africa, Kenya, and Nigeria) [14,15,16] and Asian regions (Bangladesh, Nepal, and Indonesia) [17,18,19] reported that suffering from COVID-19 was associated with food insecurity, job loss, and business closures, with coping strategies to smooth consumption. Other studies conducted in Middle Eastern countries, including Iran [20] and Pakistan [21], showed high rates of food insecurity during the initial COVID-19 lockdown. A survey conducted in India reported that household food insecurity sharply increased from 21% in December 2019 to 80% in August 2020 with a substantial worsening in diet quality [22]. Additionally, studies conducted in South American countries, including Chile [23], Brazil [24], and Peru [25], reported an increase in food insecurity levels between 2017 and 2020, especially in those with economically dependent persons (i.e., children, adolescents, and older adults).

Overall, food insecurity due to COVID-19 is strongly related to social features rather than being influenced by any governmental policy action related to a specific country. To face such an invisible burden, it has been suggested that policy recommendations should include robust, targeted social protection programs to improve access to healthy and nutritious foods, provide better protections for vulnerable and marginalized food system workers, and support resilient food production and distribution systems [26]. Government and policy control over unjustified rise in food prices is highly warranted. Finally, a commitment by physicians to screen their patients for malnutrition and food insecurity during lockdowns is further recommended.
